# Prevalence and Influencing Factors of Motoric Cognitive Risk Syndrome Among Older Adults in Long-Term Care Facilities

**DOI:** 10.3390/healthcare14172802

**Published:** 2026-09-01

**Authors:** Xinxin He, Yangyang Jiang, Langli Gao, Juan Lv, Ying Li

**Affiliations:** The Center of Gerontology and Geriatrics, West China Hospital, Sichuan University, Chengdu 610041, China; hexinxin@scu.edu.cn (X.H.); jiangyangyang@scu.edu.cn (Y.J.);

**Keywords:** motor cognitive risk syndrome, cognitive impairment, early screening, influencing factors

## Abstract

**Highlights:**

- This cross-sectional survey quantified an 8.6% MCR prevalence among older adults in long-term care facilities of Southwest China, which may indicate that slow gait exerted a stronger predictive effect on MCR onset than subjective cognitive complaints. Depressive symptoms were an independent risk factor for MCR, providing practical intervention targets for early dementia prevention in long-term care facilities.

**What are the main findings?**

- The overall prevalence of Motoric Cognitive Risk (MCR) syndrome among 730 older adults from five Southwest China long-term care facilities was 8.6%; mobility limitation may exert a stronger correlation with MCR risk than subjective cognitive complaints.
- Multivariate logistic regression suggested that elevated depressive symptoms, mild-to-moderate mobility impairment, and multimorbidity with over three chronic illnesses may act as independent risk factors for MCR after adjusting for demographic and clinical confounders.

**What are the implications of the main findings?**

- Routine combined screening for depressive symptoms, mobility function and chronic comorbidities may help identify older adults at high risk of MCR in long-term care settings, supporting early pre-dementia intervention.
- Diversified personalized activity programs may alleviate slow gait and subjective cognitive complaints, which could serve as practical preventive strategies to lower MCR incidence among institutionalized older residents.

**Abstract:**

**Background/Objectives**: To investigate the prevalence of the Motoric Cognitive Risk (MCR) Syndrome among older adults in long-term care facilities and its associated risk factors. **Methods**: From January to December 2024, older adults were recruited from five long-term care facilities in Southwest China via convenience sampling. All participants underwent a series of standardized assessments, including a self-designed demographic questionnaire, the 15-item Geriatric Depression Scale (GDS-15), the Timed Up and Go (TUG) test, the Fried frailty phenotype scale, sarcopenia assessment based on the SARC-F sarcopenia screening scale, the Basic Activities of Daily Living (BADL) scale, the Lawton Instrumental Activities of Daily Living (IADL, Lawton) scale, and the Mini-Mental State Examination (MMSE). The 4 m walk test was performed to measure participants’ gait speed. This cross-sectional study aimed to explore the prevalence of MCR syndrome and its associated influencing factors among institutionalized older adults. **Results**: The overall MCR prevalence was 8.6%. Univariate tests detected intergroup differences in BMI, depressive symptoms, hearing loss, multimorbidity, mobility impairment, frailty and several chronic diseases (all *p* < 0.05). Multivariate logistic regression analysis further demonstrated that, after adjustment for confounders, higher depressive symptom scores (OR = 1.169, 95% CI: 1.079–1.265, *p* < 0.001), mild mobility impairment (OR = 4.725, 95% CI: 1.340–16.663, *p* = 0.016), moderate mobility impairment (OR = 3.921, 95% CI: 1.146–13.420, *p* = 0.029), and more than three chronic comorbidities were independent risk factors for MCR among institutional older adults residing in long-term care facilities (OR = 2.789, 95% CI: 1.465–5.309, *p* = 0.002). **Conclusions**: Depressive symptoms, mild-to-moderate mobility impairment and multimorbidity were independent correlates of MCR among institutional older adults. Distinct risk-factor patterns existed for SG and SCCs, highlighting the separable nature of motor and subjective-cognitive dimensions. Integrated screening for emotional, physical and chronic disease risk and personalized activity interventions are warranted for long-term care residents.

## 1. Introduction

Motoric Cognitive Risk (MCR) Syndrome is defined as a clinical phenotype among older adults without dementia and intact basic activities of daily living, which is characterized by concurrent subjective cognitive complaints (SCCs) and slow gait (SG) [1]. This construct was initially proposed by Professor Joe Verghese in 2013. MCR assessment is low-cost, time-efficient and easy to conduct in communities, allowing frontline care staff to routinely screen high-risk elders and deliver early cognitive-mobility interventions.

Globally, cognitive disorders represent a growing public-health burden among older adults, with a substantial proportion of older individuals living with pre-dementia cognitive changes that confer elevated dementia risk [2]. MCR represents one important pre-dementia phenotype and has gained growing epidemiological interest. Therefore, MCR is a robust predictor of Alzheimer’s disease and vascular dementia, which can be screened in the absence of complex instruments or dedicated clinical assessments. Nevertheless, MCR prevalence varies substantially across study populations and settings. Synthesized epidemiological data demonstrate that the pooled global prevalence of MCR in older adults is 9.7%, with a pooled prevalence of 12.8% documented among Chinese older adults, a rate exceeding the pooled estimates reported in most international populations [3]. Population-based cohort studies have reported 6.1% in ELSA, 5.8% in HRS and 12.4% in CHARLS [4]. Ethnic disparity is also observed in New Zealand [5], with MCR prevalence of 4.2% among Māori older adults and 1.9% among non-Māori counterparts. Notably, existing targeted epidemiological surveys have documented that the prevalence of MCR rises sharply to 40.6% among older adults residing in long-term care facilities [6]. Currently, the exact mechanisms underlying MCR remain unclear, but most studies suggest that the onset and progression of MCR is affected by a wide array of factors relevant to older adults, encompassing physical function, residential environment and multimorbidity [7]. In institutional settings, these risk factors are highly concentrated, further accelerating both motor and cognitive decline in older adults.

Relative to non-MCR older adults, individuals presenting with MCR face substantially higher risks of adverse clinical outcomes: the risk of incident cognitive impairment and dementia rises by 120% [8], fall risk increases by 38% [9], the likelihood of hospitalization climbs by 46%, and the risk of all-cause mortality goes up by 66% [10]. Collectively, these adverse events severely compromise older adults’ quality of life and shorten their life expectancy. Institutional long-term care has emerged as a mainstream aging care model for older adults in China [11]. However, most long-term care facilities across China are situated far from urban central areas, which creates geographical barriers that greatly reduce residents’ frequency of family visits and lead to prolonged social disconnection from relatives [12]. In addition, widespread workforce understaffing constitutes a pervasive challenge within such institutional care settings. In cases where older adults develop physical dysfunction and cognitive impairment without timely standardized assessment, progressive deterioration of physical and cognitive function tends to occur. When superimposed on pre-existing chronic multimorbidity, combined physical and cognitive deficits substantially compromise the overall quality of life of residents residing in long-term care facilities.

Most existing international epidemiological investigations of MCR, covering cohorts from North America, Europe, Mexico, and South Korea, have exclusively focused on community-dwelling older adults [13,14,15]. Large-scale longitudinal and cross-sectional studies have concluded that MCR predicts dementia, recurrent falls and subsequent functional disability among non-institutional seniors. Nevertheless, dedicated systematic research targeting residents of long-term care facilities remains extremely scarce globally. In China, the available MCR literature mainly centres on urban community and rural elderly populations [2,16]; studies specifically recruiting older adults living in nursing homes or long-term care institutions are very limited, with only scattered small-sample subgroup analyses available as preliminary references. Given that MCR identification relies on brief, low-cost assessment tools suitable for routine geriatric screening, the present study aimed to estimate MCR prevalence and its multidimensional correlated risk factors among institutionalized older adults in Southwest China. Beyond analysing overall associated factors of integrated MCR syndrome, our study further explored SG and SCCs as two independent indicators separately, enabling a more refined, differentiated evaluation of motor function and self-perceived cognitive status among institutionalized older adults. Findings from this work can deliver evidence-based references for targeted early interventions addressing concurrent physical and cognitive dysfunction in this high-risk vulnerable group, as well as practically verify the operability of regular MCR screening within daily long-term care service environments.

## 2. Materials and Methods

### 2.1. Study Participants

This cross-sectional study was carried out across five long-term care facilities in Southwest China. From January to December 2024, face-to-face questionnaire interviews were completed with a total of 765 older adult residents enrolled in the survey. All participants were eligible for enrolment based on the three inclusion criteria below: (1) aged 60 years or older; (2) continuous residence in long-term care facilities for no less than 6 months; and (3) voluntary provision of written informed consent. Exclusion criteria included: (1) severe visual or auditory impairment that hindered effective communication and (2) clinical history of severe anxiety or other psychiatric disorders. After eliminating invalid questionnaires, a total of 730 participants were finally included in the present study, yielding a valid response rate of 95.4%.

### 2.2. Measures

#### 2.2.1. Assessment of MCR

The MCR criteria applied in this study were established on the basis of available literature, including subjective cognitive complaints (SCCs), slow gait (SG), preserved daily functioning, and absence of dementia [17]. (1) SCCs were assessed using the memory-related screening item extracted from GDS-15: “Do you feel that your memory is worse than that of most people?” A “yes” response to this item was classified as a positive result indicating subjective cognitive complaints. (2) SG was evaluated via the 4 m walking test to calculate participants’ gait velocity. In accordance with published references, gait velocity ≤0.8 m/s was defined as slow gait. For female participants with a height below 1.45 m, the cutoff threshold was adjusted to ≤0.66 m/s [16,18]. (3) As formal clinician interviews could not be implemented among residents in long-term care facilities in the current research, we adopted an alternative measurable criterion referenced from the Mexican Health and Aging Study. Consistent with prior relevant studies, preserved instrumental daily function was defined as no difficulty in all IADL items. The Lawton IADL scale was applied to evaluate instrumental independent functioning, given its superior performance in reflecting the long-term self-care capacity of older adults [19]. Its total score spans from 0 to 8, where a score of 8 denotes full independence in the Lawton IADL scale with no assistance required [20]. (4) Participants with an MMSE score > 26 points were defined as non-dementia individuals. This threshold was formulated by combining routine geriatric clinical experience and prior published evidence, where relevant research indicated that an MMSE value around 26 acts as an appropriate demarcation for cognitive dysfunction [21].

**Figure 1:**
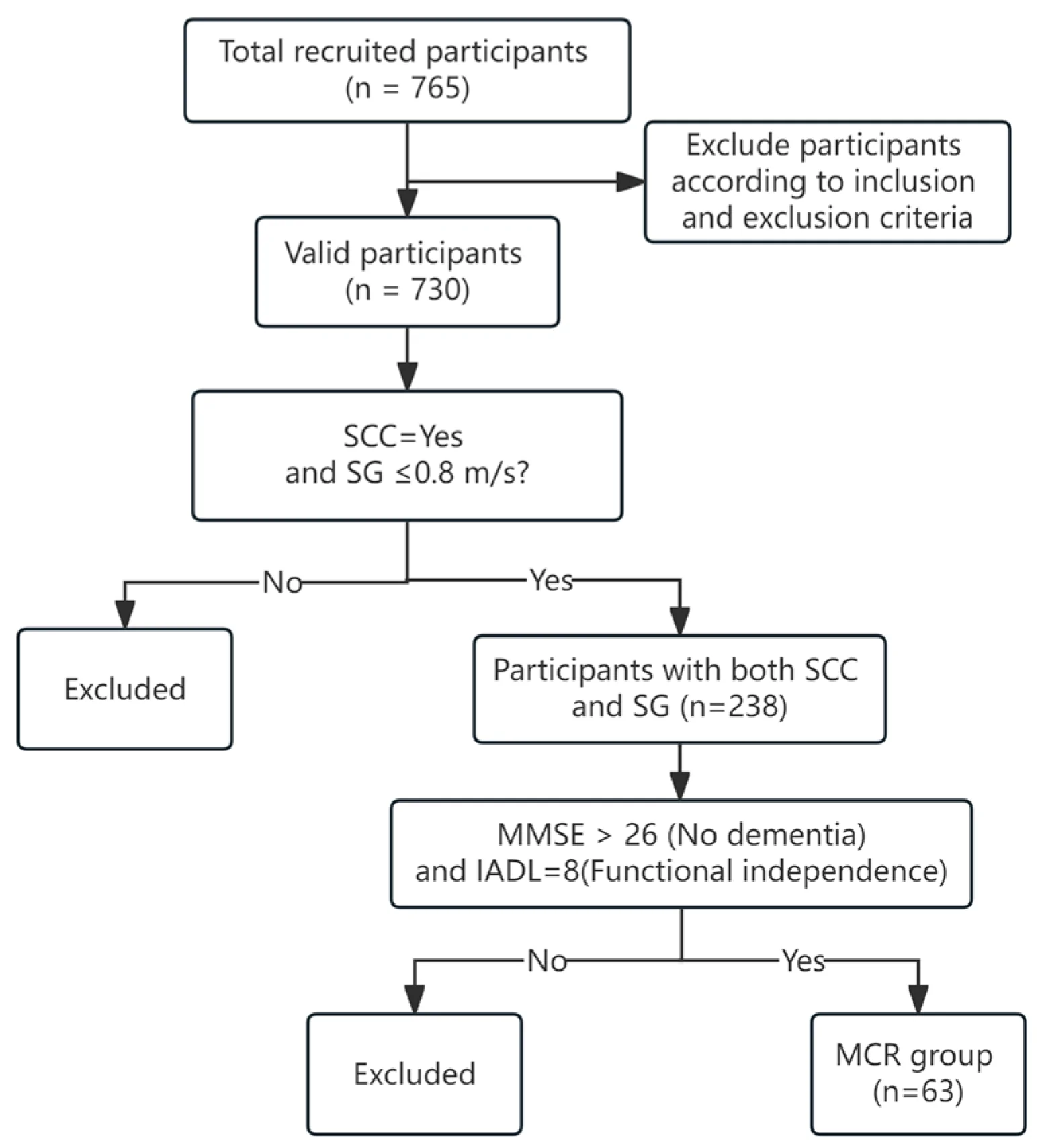
Flowchart of participant screening and subgroup classification.

To meet the complete diagnostic criteria for MCR, participants were first screened for subjective cognitive complaints (SCCs = yes) and slow gait (gait speed ≤ 0.8 m/s). A total of 238 participants satisfied these two core motor–cognitive prerequisites. We then further filtered candidates using the aforementioned MMSE cutoff (MMSE > 26) together with the criterion of full independence in instrumental activities of daily living (Lawton IADL score = 8); those who fulfilled all standards constituted the final MCR group (*n* = 63). All remaining participants who failed to meet one or more MCR criteria were assigned to the non-MCR group. The full participant screening and subgroup classification workflow is visualized in Figure 1.

#### 2.2.2. Data Collection

Data were collected via standardized face-to-face paper questionnaires. The survey instrument consisted of four primary sections: (1) Sociodemographic characteristics, including age, sex, educational attainment, marital status, and other relevant variables. Health status indicators comprised chronic medical conditions and fall history. Chronic illnesses recorded included hypertension, diabetes, stroke, malignant tumours, heart failure, asthma, arthritis, chronic pulmonary disease, renal disease, angina pectoris, and myocardial infarction. The total number of falls experienced by each long-term care resident was also documented. (2) Physical functional status: height and weight were recorded to calculate body mass index (BMI); visual and auditory function was evaluated using the Geriatric Performance Scale; and bilateral handgrip strength was tested with a hand dynamometer, and the mean value of the two measurements was computed. The Activities of Daily Living (ADL) scale was utilized to measure older adults’ daily living function. Its total score is 100 points, where a higher score corresponds to a higher level of independence [22]. The Timed Up and Go (TUG) test was adopted to evaluate physical mobility function among participants [23]. The test procedure consisted of the following sequential movements: rise from an armchair, walk forward 3 m, turn around, and return to a seated position. The total time taken to complete the full sequence was recorded. Cutoff classifications were defined as follows: a completion time of less than 10 s indicated unimpaired mobility; durations ranging from 10 to 19 s represented mild functional impairment; durations between 20 and 29 s corresponded to moderate functional impairment; and any time of 30 s or longer indicated severe functional impairment. The Fried Frailty Scale was applied to identify frailty status among participants. This scale comprises five individual components, with total scores ranging from 0 to 5. A score of 0 denotes non-frail status, while scores of 1 and above indicate the presence of frailty [24]. The SARC-F sarcopenia screening scale was adopted to evaluate sarcopenia risk among participants. The scale covers five domains: muscle strength, gait aid dependence, chair rising ability, stair climbing capacity, and fall history. Each domain is scored from 0 to 2 points; a total score of 4 and above suggests elevated sarcopenia risk [25]. (3) Lifestyle factors: current smoking status, alcohol drinking habits, and engagement in aerobic and resistance training. (4) Mental health status: Cognitive function was evaluated via the MMSE to screen for cognitive impairment among participants [26]. Depressive symptoms were measured using the 15-item Geriatric Depression Scale (GDS-15) tailored for long-term care older adults [27]. The scale contains 15 items with a maximum total score of 15, and a score of 5 and above was defined as the presence of depressive symptoms.

#### 2.2.3. Quality Control During the Investigation Process

To ensure the reliability of the research findings, all elderly long-term care facility residents were fully informed of the purpose and significance of this study before data collection, and formal investigations were initiated only after obtaining their informed consent. All investigators received standardized professional training. Specifically, the primary researcher is a senior specialist in comprehensive geriatric assessment at a Grade III Level A hospital with a valid corresponding certification, who has fully mastered the survey content and standardized assessment procedures. Paper-based questionnaires were adopted throughout the survey to guarantee complete data traceability. Upon completion of each assessment, all questionnaires were cross-checked independently by two researchers and subsequently entered into the EpiData database to maximize the accuracy of data entry.

#### 2.2.4. Statistical Analysis

All statistical analyses were performed using SPSS 21.0 software. Missing data for all research variables were checked and processed via complete case analysis due to the low missing proportion of core indicators. The normality of continuous and categorical variables was systematically examined prior to statistical testing. Normally distributed continuous data were presented as mean ± standard deviation. An independent-samples *t*-test and one-way ANOVA were applied for group comparisons of normally distributed continuous variables, whereas the Wilcoxon rank-sum test and Kruskal–Wallis H test were adopted for two-group and multi-group comparisons of non-normally distributed continuous data, respectively. Categorical and binary variables were compared using the *Pearson χ*^2^ test or Fisher’s exact test as appropriate. Prior to constructing regression models, the variance inflation factor (VIF) was calculated to assess multicollinearity among independent variables; variables with VIF > 10 were excluded from subsequent regression analyses to avoid collinearity bias. Univariate binary logistic regression was first conducted, and variables with *p* < 0.05 in univariate analyses were preliminarily included. Backward elimination was then used to establish the final multivariate binary logistic regression models for exploring the independent associations between potential influencing factors and MCR.

## 3. Results

### 3.1. Baseline Characteristics of the Study Population

A total of 730 older adults residing in long-term care facilities were enrolled in the present study, with a mean age of 83.30 ± 6.77 years. Among them, 574 participants were aged 80 years and above, accounting for 78.6% of the total sample. In terms of educational attainment, 356 participants (48.8%) had a junior high school education or lower. A total of 422 participants (57.8%) were married; 529 participants (72.5%) presented with visual impairment, whereas only 161 participants (22.1%) had hearing impairment. A total of 86.3% of older adults residing in long-term care facilities exhibited mild, moderate or severe mobility limitations during standing and walking; 46.7% were identified as frail, 22.1% met the screening criteria for sarcopenia, and 73.6% reported coughing during fluid intake. In addition, 29.3% of participants had a prior fall history. Hypertension was the most prevalent chronic comorbidity, affecting 51.7% of all subjects. The relevant data are shown in Table 1.

### 3.2. Association Between MCR and Non-MCR

We enrolled 730 older adults from long-term care facilities. The overall mean gait speed of all participants was 0.72 ± 0.45 m/s. In total, 529 residents (72.5%) met the criteria for slow gait (SG), and 341 (46.7%) reported subjective cognitive complaints (SCCs). We split the full sample into MCR and non-MCR subgroups to compare demographic, physical, cognitive and functional indicators. Sixty-three participants (8.6%) were categorized as having MCR, and the remaining 667 (91.4%) formed the non-MCR group.

Compared with non-MCR participants, those with MCR were slightly older (84.02 ± 5.83 vs. 83.24 ± 6.86 years) and had slower gait speed (0.58 ± 0.15 vs. 0.65 ± 0.27 m/s), consistent with the core diagnostic features of MCR. For cognitive performance measured via the MMSE, the MCR group had a higher average score (27.79 ± 1.23) than the non-MCR group (20.42 ± 6.30). Regarding daily function, MCR participants scored marginally higher on both BADL (94.44 ± 9.07 vs. 93.56 ± 11.16) and Lawton IADL (6.61 ± 1.75 vs. 5.99 ± 1.97). Full stratified comparisons between the two groups are summarized in Table 2.

### 3.3. Exploring Influencing Factors of MCR via Multivariate Logistic Regression

Baseline demographic, physical, lifestyle and mental characteristics were compared between the MCR group and the non-MCR group. Compared with participants in the non-MCR group, those with MCR exhibited significant intergroup differences (all *p* < 0.05) in the Timed Up and Go test results, depressive symptom scores, Lawton IADL scores, number of chronic comorbidities, height, BMI, MMSE scores, hearing impairment status, and frailty status, as well as the prevalence of stroke, heart failure, arthritis and chronic obstructive pulmonary disease (COPD).

Binary logistic regression analysis was performed with MCR status set as the dependent variable (0 = non-MCR, 1 = presence of MCR). All variables showing statistically significant intergroup differences in Table 1 were entered into the regression model. Multivariate stepwise logistic regression was adopted, with age, educational attainment, marital status, BMI, current smoking, alcohol intake and fall history adjusted as confounding covariates.

The results revealed that depressive symptom scores, the number of chronic comorbidities (≤3 vs. >3), and Timed Up and Go test performance were independent risk predictors for MCR among older adults residing in long-term care facilities (all *p* < 0.05). The Nagelkerke *R*^2^ of the final regression model was 0.159. The Hosmer–Lemeshow goodness-of-fit test demonstrated satisfactory model calibration: *χ*^2^ = 6.135, *df* = 7, and *p* = 0.524 (*p* > 0.05). Detailed regression outputs are presented in Table 3.

### 3.4. Multivariate Logistic Regression Analysis of Factors Associated with SG

Since the predictors of MCR among older adults in long-term care facilities identified in the present study showed slight discrepancies with those reported in prior research, and only a limited number of significant correlates were observed for the composite MCR outcome, we decomposed MCR into SG and SCCs and performed separate regression analyses to identify the respective independent risk factors for SG and SCCs in long-term care facility residents.

Based on the diagnostic definitions provided in the Methods, SG was defined as a walking speed of ≤0.8 m/s, with participants coded as one for SG and zero for non-SG. Univariate analysis showed that age group, educational level, hearing status, standing and walking ability, frailty, sarcopenia, depression, resistance exercise, aerobic exercise, diabetes, stroke, heart failure, asthma, comorbidity count, and marital status exhibited statistically significant associations with SG (all *p* < 0.05).

Variables with statistically significant correlations with SG from univariate analyses were included in a binary logistic regression model to explore the independent risk factors for SG. The multivariate results demonstrated that age group, standing and walking ability, resistance exercise, diabetes, and asthma served as independent risk factors for SG (*p* < 0.05). According to the results presented in Table 4, relative to the 60–69-year age group, older adults aged 70–79 years and ≥80 years had 3.100-fold and 3.948-fold higher risks of SG, respectively. Standing and walking ability exerted an overall significant effect on SG incidence. Compared with the reference group with normal walking speed, moderate impairment and severe walking impairment were linked to substantially reduced SG risks (*OR* = 0.048 and 0.005, respectively). Residents with resistance exercise habits, diabetes mellitus, or asthma showed 2.935, 1.938 and 2.907 times higher odds of SG than those without the corresponding conditions. The Hosmer and Lemeshow test of the final multivariate model showed a chi-square value of 4.098 with eight degrees of freedom (*p* = 0.848), indicating excellent goodness of fit. The Nagelkerke *R*^2^ of the full model was 0.474, which suggested that the incorporated predictors could explain 47.4% of the variance in the study outcome. (*χ*^2^ = 4.098, *df* = 8, and *p* = 0.848).

### 3.5. Exploring Factors Influencing SCCs Through Multivariate Logistic Regression

Univariate analysis showed statistically significant associations between SCCs and gender, educational level, visual acuity, hearing status, frailty, depression, fatigue, hypertension, stroke, heart failure, asthma, arthritis, COPD, angina, myocardial infarction, number of comorbidities, and marital status (*p* < 0.05).

Multivariate binary logistic regression analysis demonstrated that gender, hearing status, depression stratification, and the count of chronic comorbidities were independent risk factors for SCCs (all *p* < 0.05), and detailed outputs are presented in Table 5. Compared with men, women had 1.536-fold higher odds of SCCs. Hearing impairment served as a protective factor, with an OR of 0.598 versus participants without hearing impairment. Depression stratification as a whole was significantly associated with SCCs. Only severe depression yielded an independent protective effect (*OR* = 0.395, 95% CI: 0.263–0.594), while mild and moderate depression failed to reach statistical significance as independent predictors. Individuals living with multiple chronic conditions exhibited a 3.199-fold higher risk of SCCs compared to those with limited or no chronic diseases. The Hosmer and Lemeshow test for the final multivariate model yielded a chi-square value of 2.019 with five degrees of freedom (*p* = 0.847), indicating satisfactory model fit. The Nagelkerke *R*^2^ of the final model was 0.143, suggesting that the predictors included in the model explained 14.3% of the variance in SCC status.

## 4. Discussion

All data were collected through face-to-face on-site surveys conducted within participating long-term care facilities. Our findings showed that the overall MCR prevalence among institutionalized older adults in Southwest China was 8.6%. This figure was markedly lower than the 40.6% reported by Wang X et al. [6] in a Chinese institutional sample, yet higher than the 7.8% prevalence documented among geriatric outpatients in a Turkish cohort study [28]. Prior large-cohort studies have documented MCR prevalence ranging from 5.8% to 17.4% [2,14,29]. Such discrepancies likely arise from two major sources of heterogeneity: differences in sample geography and ethnic makeup, as well as inconsistent diagnostic cutoffs used to define MCR. Notably, the MCR group exhibited a higher mean MMSE score than the non-MCR group in our study. This seemingly counterintuitive observation is a direct consequence of the strict MCR diagnostic criteria adopted in this research. Consistent with the classic MCR definition, only older adults without dementia (MMSE > 26) and fully preserved instrumental activities of daily living were eligible to be classified into the MCR group. As documented previously, older adults residing in long-term care facilities carry a heavier burden of cognitive dysfunction and tend to obtain lower MMSE scores compared with community-dwelling older adults [30]. In our sample, many institutional residents with obvious cognitive impairment or dementia did not meet MCR eligibility criteria and were classified into the non-MCR group, which substantially reduced the average MMSE score of this subgroup. Although individuals with MCR maintain intact global cognitive function, reflected by normal MMSE at baseline, the coexistence of SCCs and SG confers elevated risk of subsequent cognitive deterioration. Hence, early screening and targeted interventions for MCR populations in long-term care settings are of great clinical value to delay the onset of dementia.

In terms of the selection of cognitive assessment instruments for MCR identification, Verghese originally proposed the MMSE to assess cognitive status for MCR diagnosis in 2013 [17]; yet this tool is vulnerable to confounding factors including age, education level, gait impairment, and visual loss [31]. In contrast, Wang et al. [6] adopted the Ascertain Dementia 8-item Questionnaire (AD8) Dementia Screening Interview for the institutional cohort in this study. Although the AD8 allows quick identification of early cognitive decline, the screening instrument bears a false-positive rate as high as 50% [32]. Adding to these measurement-related differences, participants recruited from the long-term care facilities in this study were predominantly advanced-age older adults, with a mean age exceeding 80 years. Published data show that cognitive impairment is far more prevalent within this extreme age group [33], which inherently restricted our available sample pool. To minimise confounding bias arising from these demographic and behavioural differences, we adjusted all statistical models for the following covariates: age, educational level, marital status, body mass index, current smoking status, regular alcohol consumption, and fall history. This comprehensive multivariable adjustment helps reduce residual confounding and strengthens the internal validity of our findings.

Logistic regression analyses revealed that depressive symptom scores constituted an independent risk factor for MCR among long-term care residents following full adjustment for all pre-defined confounders (*OR* = 1.169,95%CI: 1.079–1.265, *p* < 0.001). Specifically, each one-point elevation in depressive symptom scores corresponded to a 16.9% higher odds of exhibiting MCR. Consistent with prior evidence, elevated depressive symptoms diminish long-term care residents’ motivation to participate in outdoor and social activities [34]. Reduced engagement further lowers overall activity levels, thereby intensifying loneliness and anxious affect [35]. Meanwhile, persistent negative mood states alter cerebral neurotransmitter secretion, which in turn accelerates the progression of cognitive deterioration. Furthermore, chronic depressive symptoms are strongly correlated with incident MCR [36]. Regardless of the trajectory—persistent, progressively worsening, or remitted following severe prior episodes—prolonged exposure to negative affective states elevates the risk of MCR emergence during the pre-dementia phase [37]. Accordingly, great attention must be paid to comprehensive psychological assessment and timely emotional intervention for long-term care older adults during early cognitive screening. Apart from standardized mood scale assessments, long-term care facilities ought to carry out multiple supportive measures: organizing regular social interaction and outdoor exercise programmes to reduce loneliness, delivering one-on-one psychological guidance, training nursing caregivers to identify early depressive signals, and establishing a collaborative referral pathway with geriatric psychiatrists for residents with prolonged depressive symptoms. Such combined mental health strategies may effectively lower the risk of subsequent MCR.

Comorbidity analysis showed that older adults with more than three chronic conditions had a 2.789-fold higher risk of MCR compared to those with three or fewer conditions (*OR* = 2.789, 95% CI: 1.465–5.309, and *p* = 0.002). Multimorbidity was also an independent risk factor for the presence of SCCs. Consistent with the univariate analysis, stroke, heart failure, malignant tumours, arthritis, and COPD were each significantly associated with the prevalence of MCR among older adults in long-term care facilities (*p* < 0.05). Consistent with the findings of the meta-analysis by Jayakody et al. [38], which encompassed 21 countries, stroke and cardiac conditions were identified as risk factors for MCR. By contrast, associations between hypertension or diabetes and MCR vary across countries and regions. This heterogeneity may partly stem from differences in medication adherence among affected patients. Our data do not support a strong association between hypertension or diabetes and MCR prevalence. A potential explanation is that older adults in long-term care often have better medication adherence than those living in the community. Taken together, our findings suggest that early MCR screening should be prioritised for long-term care residents with multiple chronic conditions, alongside routine efforts to improve medication adherence among people living with chronic illness.

We included the standing-to-walking test as an independent variable in the multivariate logistic regression model, with unimpaired standing-to-walking as the reference category. Under this model, mild impairment (*OR* = 4.725, 95%CI: 1.340~16.663, and *p* = 0.016) and moderate impairment (*OR* = 3.921, 95%CI: 1.146~13.420, and *p* = 0.029) in this task appeared to be independently associated with MCR among older adults residing in long-term care facilities. The standing-to-walking test is widely used to assess lower-limb strength, dynamic balance, and fall risk in older adults, and has been regarded as a sensitive measure of subclinical motor decline [39].

There were no significant differences in ADL or Lawton IADL scores between the MCR and non-MCR groups, suggesting that long-term care residents with MCR had not yet developed meaningful deficits in basic or instrumental activities of daily living, and instead exhibited only mild-to-moderate impairments in motor function and balance. Neither aerobic nor resistance exercise showed a statistically significant association with MCR in this study (*p* > 0.05), which is inconsistent with the findings reported by Ambrose et al. [40]. A possible explanation for this discrepancy is that physical activity spaces in long-term care facilities are often limited, and residents may perceive the provided aerobic and resistance exercises as routine, monotonous, low-intensity activities uniformly organized by facility staff—quite different from the multicomponent and individualized exercise intervention protocols described in previous studies. On this basis, greater attention could be given to interventions targeting lower-limb strength and balance function in older adults with mild-to-moderate standing-to-walking difficulties [41]. Moreover, multicomponent exercise strategies might be more widely implemented in long-term care facilities, alongside individualized aerobic and resistance training, to help reduce the prevalence of MCR among institutionalized older adults. Older adults with MCR tend to have weaker lower-limb strength and may therefore be at increased risk of falls. Although people with MCR typically have reduced lower-limb strength and consequently higher fall risk, severe stand-to-walk impairment was not significantly associated with MCR in our analysis. This finding may partly reflect safety-driven exclusion of participants with severe functional impairment during assessments. The resulting small sample within this subgroup likely reduced statistical power and hindered our ability to detect a significant association.

We performed separate multivariate logistic regression analyses for the two core components of MCR, namely slow gait (SG) and subjective cognitive complaints (SCCs). Our component-specific regression outputs provide empirical clues regarding the interrelated nature of SG and SCCs among institutionalized older adults. Physical dysfunction manifested by decreased walking speed may restrict social participation and limit access to cognitive stimulation, which in turn aggravates memory deterioration and subjective cognitive concerns. Collectively, SG and SCCs may co-occur and contribute jointly to MCR development among long-term care residents.

The present study has several notable strengths with clear clinical relevance. We focused on long-term care residents, a high-risk group that remains under-represented in global MCR research, where most existing evidence originates from community-dwelling populations. Rather than only analysing the composite MCR endpoint, separate multivariate analyses for SG and SCCs allow us to disentangle heterogeneous risk patterns for motor-related impairment and self-perceived cognitive concerns, yielding a more nuanced understanding of MCR in residential aged-care settings. Moreover, all screening tools used in this study are simple and feasible for routine geriatric assessment. Our findings on modifiable risk factors support integrated screening and targeted risk-reduction strategies applicable to long-term care facility practice.

Nevertheless, several important limitations should be acknowledged. As a cross-sectional observational study, causal inferences cannot be drawn, only correlational associations can be established for MCR and its two components. In addition, SCCs were evaluated via one single item from the GDS-15 instead of a dedicated cognitive-complaint scale. Though this single-item method has been used in prior literature, the item is derived from a depression-screening tool and is vulnerable to mood-related measurement confounding, which warrants cautious interpretation of associations involving SCCs. Several potential geriatric risk factors including nutritional status, social support and medication information were not collected in our study, and these unmeasured variables may also contribute to MCR-related phenotypes. Furthermore, relatively low Nagelkerke *R*^2^ values across our regression models are typical for multifactorial geriatric-syndrome observational studies, indicating that our included predictors can only partially explain outcome variance.

Future research could adopt multi-site sampling covering wider age ranges and diverse social backgrounds. Key variables such as nutrition, social support and medication profiles should be incorporated to construct more comprehensive analytical models. Longitudinal study designs are also essential to clarify temporal sequences and potential causal pathways among MCR-related risk factors, SG, and SCCs.

## 5. Conclusions

In this cross-sectional study of 730 long-term care residents in Southwest China, depression, multimorbidity, and stand-to-walk impairment were identified as important correlates of MCR. Separate analyses of the two core MCR components further revealed divergent risk-factor patterns: certain factors were predominantly associated with SG, while depression was more closely linked to SCCs. These findings underscore the need for integrated management addressing emotional well-being, physical function, and chronic disease burden among long-term care residents, to enable early intervention for motor–cognitive vulnerability.

## Figures and Tables

**Table 1:** Comparison of general characteristics between participants with MCR and the non-MCR group.

Items	MCR	No-MCR	χ^2^/*Z*	*p*	Items	MCR	No-MCR	χ^2^/*Z*	*p*
Age, years	84.02 ± 5.83	83.24 ± 6.86	−0.894	0.372	Gender				
Height, cm	152.66 ± 6.70	154.89 ± 8.41	−2.057	0.040	Male	15 (23.8)	215 (32.2)	1.893	0.169
Body weight, kg	56.56 ± 7.47	56.15 ± 10.63	−0.611	0.541	Female	48 (76.2)	452 (67.8)		
BMI	24.28 ± 2.99	23.32 ± 3.57	−2.181	0.029	TUG impairment grade				
Calf girth, cm	32.93 ± 2.51	32.65 ± 3.49	−0.498	0.619	No impairment	0 (0.0)	100 (15.0)	18.097	<0.001
Handgrip, kg	17.09 ± 6.13	18.26 ± 6.88	−1.212	0.226	Mild impairment	38 (60.3)	343 (51.5)		
Lawton IADL	6.61 ± 1.75	5.99 ± 1.97	−0.767	0.443	Moderate impairment	22 (34.9)	141 (21.1)		
ADL	94.44 ± 9.07	93.56 ± 11.16	−0.648	0.517	Severe impairment	3 (4.8)	83 (12.4)		
GDS-15	4.67 ± 2.99	2.75 ± 2.93	−5.688	<0.001	Frailty				
comorbidity					No	26 (41.3)	363 (54.4)	4.001	0.045
≤3	46 (73.0)	612 (91.8)	22.732	<0.001	Yes	37 (58.7)	304 (45.6)		
>3	17 (27.0)	55 (8.2)			Sarcopenia				
Education					No	46 (73.0)	523 (78.4)	0.975	0.324
Middle school and below	35 (55.6)	321 (48.1)	2.361	0.501	Yes	17 (27.0)	144 (21.6)		
High school	17 (27.0)	190 (28.5)			Depression grade				
College	9 (14.3)	105 (15.7)			No depression	33 (52.4)	517 (77.5)	19.608	<0.001
Bachelor and above	2 (3.1)	51 (7.7)			Mild depression	23 (36.5)	111 (16.6)		
Smoking					Moderate depression	4 (6.3)	29 (4.4)		
Yes	7 (11.1)	95 (14.2)	0.470	0.493	Severe depression	3 (4.8)	10 (1.5)		
No	56 (88.9)	572 (85.8)			Fatigue				
Marital status					Yes	26 (41.3)	197 (29.5)	3.736	0.053
With spouse	26 (41.3)	395 (59.2)	0.006	0.940	No	37 (58.7)	470 (70.5)		
Without spouse *	37 (58.7)	272 (40.8)			Resistance exercise				
Alcohol drinking					Yes	18 (28.6)	162 (24.3)	0.569	0.451
Yes	5 (7.9)	78 (11.7)	0.807	0.369	No	45 (71.4)	505 (75.7)		
No	58 (92.1)	589 (88.3)			Aerobic exercise				
Visual impairment					Yes	3 (4.8)	63 (9.4)	1.535	0.215
Yes	40 (63.5)	489 (73.3)	2.783	0.095	No	60 (95.2)	604 (90.6)		
No	23 (36.5)	178 (26.7)			Hypertension				
Hearing impairment					Yes	39 (61.9)	339 (50.8)	2.830	0.092
Yes	21 (33.3)	140 (21.0)	5.102	0.024	No	24 (38.1)	328 (49.2)		
No	42 (66.7)	527 (79.0)			Diabetes				
Malignant tumour					Yes	17 (27.0)	137 (20.5)	1.436	0.231
Yes	4 (6.3)	13 (1.9)	4.900	0.027	No	46 (73.0)	530 (79.5)		
No	59 (93.7)	654 (98.1)			Stroke				
Heart failure					Yes	10 (15.9)	51 (7.6)	5.087	0.024
Yes	9 (14.3)	35 (5.2)	8.302	0.004	No	63 (84.1)	616 (92.4)		
No	54 (85.7)	632 (94.8)			Asthma				
Osteoarthritis					Yes	4 (6.3)	51 (7.6)	1.000	0.475
Yes	29 (46.0)	205 (30.7)	6.185	0.013	No	59 (93.7)	616 (92.4)		
No	34 (54.0)	462 (69.3)			Kidney disease				
COPD					Yes	10 (15.9)	79 (11.8)	0.873	0.350
Yes	13 (20.6)	78 (11.7)	4.217	0.04	No	53 (84.1)	588 (88.2)		
No	50 (79.4)	589 (88.3)			Myocardial infarction				
Angina pectoris					Yes	1 (1.6)	14 (2.1)	0.075	0.784
Yes	10 (15.9)	69 (10.3)	1.823	0.177	No	62 (98.4)	653 (97.9)		
No	53 (84.1)	598 (89.7)			Aspiration				
Fall history					Yes	40 (63.5)	497 (74.5)	3.595	0.058
Yes	18 (28.6)	196 (29.4)	0.018	0.892	No	23 (36.5)	170 (25.2)		
No	45 (71.4)	471 (70.6)							

* Without spouse included unmarried, divorced and widowed participants.

**Table 2:** Comparison between participants with and without MCR.

Items	MCR	non-MCR
SCCs (*n*, %)	63 (100%)	0 (0%)
SG (mean ± SD, m/s)	0.58 ± 0.15	0.65 ± 0.27
MMSE (mean ± SD)	27.79 ± 1.23	20.42 ± 6.30
Lawton IADL (mean ± SD)	6.61 ± 1.75	5.99 ± 1.97

**Table 3:** Multivariate logistic regression analysis of factors associated with MCR.

Independent Variable	*b*	*SE*	Wald *χ*^2^	*p*	*OR* (95%CI)
GDS-15	0.156	0.041	14.723	<0.001	1.169 (1.079, 1.265)
number of chronic comorbidities (reference group: ≤3 chronic conditions)					
>3 chronic conditions	1.026	0.329	9.746	0.002	2.789 (1.465, 5.309)
Timed Up and Go test performance (reference group: no mobility impairment)					
mild impairment	1.553	0.643	5.832	0.016	4.725 (1.340, 16.663)
moderate impairment	1.366	0.628	4.739	0.029	3.921 (1.146, 13.420)
severe impairment	−17.265	3980.816	0.000	0.997	0.000 (0.000, —)
constant	−4.254	0.647	43.289	<0.001	0.014

**Table 4:** Multivariate logistic regression analysis of factors influencing SG.

Independent Variable	*b*	*SE*	Wald *χ*^2^	*p*	*OR* (95%CI)
age group (reference: 60–69 y)			10.580	0.014	/
60–69 y	0.631	0.476	1.755	0.185	1.879 (0.739, 4.778)
70–79 y	1.132	0.437	6.708	0.010	3.100 (1.317, 7.300)
≥80 y	1.373	0.521	6.949	0.008	3.948 (1.422, 10.960)
Timed Up and Go test performance (reference group: no mobility impairment)			95.347	<0.001	
mild impairment	−0.414	1.135	0.133	0.715	0.661 (0.071, 6.109)
moderate impairment	−3.046	1.025	8.830	0.003	0.048 (0.006, 0.355)
severe impairment	−5.239	1.057	24.547	<0.001	0.005 (0.001, 0.042)
resistance exercise	1.077	0.365	8.695	0.003	2.935 (1.435, 6.004)
diabetes	0.661	0.272	5.910	0.015	1.938 (1.137, 3.303)
asthma	1.067	0.490	4.742	0.029	2.907 (1.113, 7.596)
constant	2.567	1.109	5.356	0.021	13.027

**Table 5:** Multivariate logistic regression analysis of factors influencing SCCs.

Independent Variable	*b*	*SE*	Wald *χ2*	*p*	*OR* (95%CI)
Gender (reference: male)	0.429	0.172	6.194	0.013	
Hearing (reference: no hearing impairment)	−0.514	0.193	7.072	0.008	
Number of chronic comorbidities (reference group: ≤3 chronic conditions)	1.163	0.294	15.629	<0.001	3.199 (1.797, 5.692)
Constant	0.096	0.366	0.068	0.794	1.100 (—, —)

## Data Availability

Restrictions apply to the sharing of raw questionnaire data to protect the confidentiality of human participants in accordance with the approved ethical protocol. The anonymized questionnaire dataset has been uploaded only for internal editorial review. Qualified researchers may submit formal applications to the corresponding author to obtain relevant datasets.

## Abbreviations

The following abbreviations are used in this manuscript:

MCR	Motoric Cognitive Risk
SG	Slow Gait
SCCs	Subjective Cognitive Complaints
GDS-15	15-item Geriatric Depression Scale
TUG	Timed Up and Go Test
SARC-F	Strength, Assistance with Walking, Rise from a Chair, Climb Stairs, Falls Screening Tool
BADL	Basic Activities of Daily Living
IADL	Instrumental Activities of Daily Living
MMSE	Mini-Mental State Examination
OR	Odds Ratio
CI	Confidence Interval
